# 1035. Manufacturing Processes of SER-109, a Purified Investigational Microbiome Therapeutic, Reduce Risk of Transmission of Emerging and Undetected Infections in Donor Stool

**DOI:** 10.1093/ofid/ofab466.1229

**Published:** 2021-12-04

**Authors:** Christopher McChalicher, Ahmad Abdulaziz, Elizabeth Halvorsen, Mary-Jane Lombardo, Jonathan Winkler, Sanabel Almomani, Barbara McGovern, Gregory McKenzie, David Ege, John Aunins

**Affiliations:** 1 Seres Therapeutics, Cambridge, Massachusetts; 2 Seres Therapeutics, Inc, Cambridge, Massachusetts; 3 Seres Therapeutics, Inc., Cambridge, MA; 4 Seres Therapeutics (Current: Prolacta Biosciences), Cambridge, Massachusetts

## Abstract

**Background:**

Fecal microbiota transplantation (FMT) is vulnerable to emerging pathogens due to reliance on donor screening for risk mitigation. These concerns were highlighted by dual FDA safety alerts regarding FMT transmission of bacterial pathogens, which were recognized in hindsight only after hospitalizations and deaths. The FDA also warned of potential risk of SARS-CoV-2 transmission, leading to quarantine of FMT in March 2020, two months after COVID-19 was reported on US soil. Conversely, our development program for SER-109, an oral investigational microbiome therapeutic, was prospectively designed to inactivate organisms of concern, while purifying the hardy Firmicutes spores. We evaluated whether the manufacturing processes for SER-109 inactivate model organisms, including a coronavirus with gastrointestinal tropism, and a representative Gram-negative bacterium.

**Methods:**

Model organisms were selected based on biologic suitability, detectability, and laboratory safety. Porcine Epidemic Diarrhea Virus (PEDV, a coronavirus) was selected to model SARS-CoV-2. Quantitation used a Vero cell tissue culture infectious dose (TCID_50_) assay. For *E. coli*, a rifampicin-tolerant *Salmonella enterica* was selected and quantified with MacConkey lactose agar plus rifampicin. Spiking experiments into representative fecal suspensions were completed to measure inactivation of model organisms. Log-reduction factors (LRF) were calculated based on the drop in organism titer during inactivation. Hold controls in non-ethanolic test matrices were used to confirm specificity of the ethanol inactivation.

**Results:**

In 70% v/v ethanol, PEDV was inactivated by more than 4.2 log_10_ (to limit of detection, LOD) within 4 minutes (Fig1). In 50% v/v ethanol, *S. enterica* was inactivated by more than 6.5 log_10_ (to LOD) within 30 seconds (Fig2).

Figure 1. Inactivation of Porcine Epidemic Diarrhea Virus (PEDV), log10 reduction factor (LRF) versus time

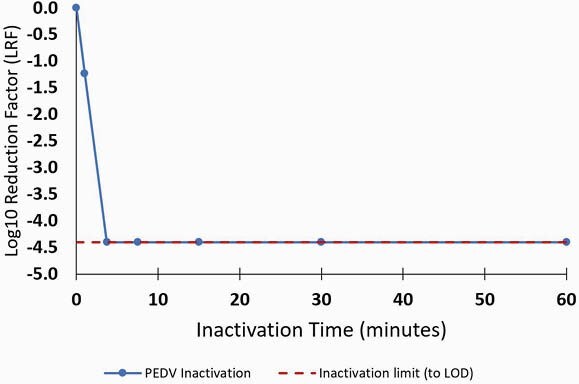

Average of two experiments shown. Also shown is the maximum achievable inactivation based on the limit of detection (LOD).

Figure 2. Inactivation of S. enterica, log10 reduction factor (LRF) versus time.

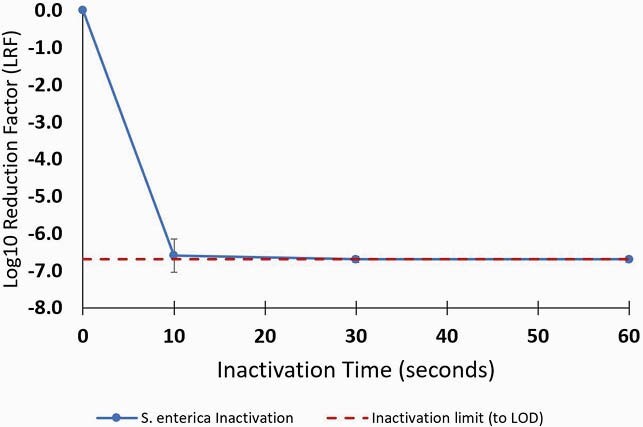

Average of three experiments with error bars represent 95% CI. Also shown is the maximum achievable inactivation based on the limit of detection (LOD).

**Conclusion:**

These experiments demonstrate substantial inactivation of the model organisms and support the potential benefit of SER-109 manufacturing process to mitigate risks of undetected or emerging pathogens for which reliable screening is limited. Ethanol exposure leads to a purified investigational product of beneficial Firmicutes spores while affording a safety net beyond donor screening alone.

**Disclosures:**

**Christopher McChalicher, n/a**, **Seres Therapeutics** (Employee, Shareholder) **Ahmad Abdulaziz, MS**, **Seres Therapeutics Inc.** (Employee, Shareholder) **Elizabeth Halvorsen, PhD**, **Seres Therapeutics** (Employee, Shareholder) **Mary-Jane Lombardo, PhD**, **Seres Therapeutics** (Employee, Shareholder) **Jonathan Winkler, PhD**, **Seres Therapeutics** (Employee, Shareholder) **Barbara McGovern, MD**, **Seres Therapeutics** (Employee, Shareholder) **Gregory McKenzie, PhD**, **Prolacta Bioscience** (Employee) **David Ege, PhD**, **Merck & Co., Inc.** (Shareholder)**Seres Therapeutics** (Employee, Shareholder) **John Aunins, PhD**, **Seres Therapeutics, Inc.** (Employee)

